# Proteolytic Activity of Silkworm Thorn (*Cudrania tricuspidata*) Fruit for Enzymatic Hydrolysis of Food Proteins

**DOI:** 10.3390/molecules29030693

**Published:** 2024-02-02

**Authors:** Na-Eun Yang, Da-Hoon Lee, Jun Hwang, Woo-Young Son, Kyeong-Soo Kim, Gwang-Yeon Kim, Hyun-Wook Kim

**Affiliations:** 1 Department of GreenBio Science, Gyeongsang National University, Jinju 52725, Republic of Korea; ly2223@naver.com; 2 Division of Animal Bioscience & Integrated Biotechnology, Gyeongsang National University, Jinju 52828, Republic of Korea; ldh0766@naver.com (D.-H.L.); hwangjun1116@naver.com (J.H.); sonwy001223@naver.com (W.-Y.S.); 3 Department of Pharmaceutical Engineering, Gyeongsang National University, Jinju 52725, Republic of Korea; soyoyu79@gnu.ac.kr; 4 Sancheong Hanbang Kkujippong Farming Association Corporation, Sancheong 52255, Republic of Korea; yeone69@hanmail.net

**Keywords:** bioactive peptide, enzymatic hydrolysis, protein hydrolzsate, plant protease, proteolysis

## Abstract

This study aimed to isolate the proteolytic fraction from the silkworm thorn fruit (*Cudrania tricuspidata*) through ethanol precipitation at different ratios, and to determine its proteolytic activity and optimal activity conditions. Furthermore, the hydrolysis characteristics and antioxidant activity of soy protein isolate (SPI) and whey protein concentrate (WPC) hydrolyzates obtained through the enzymatic hydrolysis of freeze-dried silkworm thorn fruit powder (SF) were evaluated. For isolation and partial purification of proteolytic fraction, the water-solubilized fraction of the silkworm thorn fruit was purified through ethanol precipitation at four different ratios of 1:1, 1:2, 1:4, and 1:6 (*v/v*). The protein recovery rate, caseinolytic activity, protein pattern, and optimal activity (pH, temperature, and inhibitors) of fractional ethanol precipitate obtained from the silkworm thorn fruit (ESF) were evaluated. The proteolytic fraction obtained from silkworm thorn fruit exhibited a major protein band around 65–70 kDa and showed the highest proteolytic activity at a 1:4 ratio of ethanol precipitation (*p* < 0.05). The optimal activity of the measured enzyme fraction was determined to be at pH 9.0 and 50 °C, and the proteolytic activity of ESF was almost inhibited by phenyl methyl sulphonyl fluoride (PMSF, 2 mM), a serine protease inhibitor. Compared to Alcalase and papain, extensively used as commercial enzymes, the silkworm thorn fruit powder was less effective in hydrolyzing SPI and WPC. Nevertheless, SPI and WPC hydrolyzates mediated with silkworm thorn fruit powder showed even better antioxidant activities than those mediated with Alcalase and papain. Thus, our results show the potential application of silkworm thorn fruit as a novel source of plant protease for producing human-grade protein hydrolyzates.

## 1. Introduction

Human-grade protein hydrolyzate, primarily obtained through enzymatic hydrolysis of food proteins, has been extensively utilized in the food processing industry to improve absorption rates, physiological functionality (e.g., antioxidant and antihypertensive activities), palatability, and solubility. Additionally, enzymatic hydrolysis can reduce the allergenicity of processed foods [1,2]. In the enzymatic hydrolysis process, the selection of a protease not only affects hydrolysis efficiency but also tremendously impacts the production of peptides and amino acids, owing to the different selective peptide cleavage sites of each proteolytic enzyme [3]. In practice, various proteolytic enzymes are employed in the production of bioactive peptides, sourced from animals (typically pepsin and trypsin), plants (papain and bromelain), and microorganisms (subtilisin-type enzymes-Alcalase, Neutrase, and Pronase) [4]. Although microbial enzymes have been used industrially for reasons such as reducing production costs and enabling large-scale production, plant-derived proteolytic enzymes have the advantage of a wide optimal range of temperatures and pH [5]. Moreover, plant proteases are less likely to cause allergic reactions than microbial enzymes and can contribute to the formation of unique flavors in fermented meat and dairy products [6]. Thus, exploring and applying novel proteolytic enzymes from plant sources still has important implications for the food industry.

Silkworm thorn (*Cudrania tricuspidata*) is a mulberry tree that grows naturally in East Asia, and its stem, leaves, and fruit are noteworthy food and herbal medicine ingredients in Korea. Several physiological benefits of silkworm thorn fruit have been previously reported, such as antihypertensive activity [7], antioxidant activity [8], hepatoprotective effect [9], and neuroprotective effect [10]. The promising physiological activity of silkworm thorn fruit could be related to mitigating oxidative stress through polyphenols and crude polysaccharides [8,10]. The cultivation of silkworm thorns in a commercial production farming system for use as a health-promoting food ingredient has recently been expanding in Korea. Thus, silkworm thorn fruit has promising value as a food ingredient that provides a positive perception to consumers, and it could be expected that a recent increase in production through large-scale farms can secure price merit. However, there have been limited studies on using silkworm thorn fruit as a source for isolating plant-derived proteolytic enzymes. It has been documented that proteolytic enzymes exist in the roots, stems, leaves, and fruits of Moraceae trees, and previous studies have identified macluralisin, a serine protease, in the fruits of Osage orange (*Maclura pomifera*) of the Moraceae family [11,12]. Macluralisin (molecular weight of 65 kDa) is a subtilisin-like serine protease with optimal activity at pH 8.5 and 58 °C [11]. In addition, Kim et al. [13] recently reported the proteolytic activity of silkworm thorn fruit on major skeletal muscle proteins in chicken breast, including the myosin heavy chain. However, no literature has reported the proteolytic activity of silkworm thorn fruit on conventional food proteins used in manufacturing human-grade protein hydrolyzate, such as soy and milk proteins.

Ethanol precipitation, a practical and simple method, is advantageous for separating and purifying proteinous compounds, including enzymes, from plant sources due to its ability to selectively precipitate proteins, since it could be effective in removing contaminants such as pigments and polysaccharides [14]. Previously, it has been noted that peptidases from the latex of Maclura pomifera fruits have been successfully isolated through the ethanol precipitation method [15]. Taken together, proteolytic enzymes, including a serine-type macluralisin, exist in silkworm thorn fruit and could be expected as a novel proteolytic enzyme for producing bioactive peptides. Therefore, this preliminary study aimed to isolate the proteolytic fraction from silkworm thorn fruit, through ethanol precipitation at different ratios and to determine its proteolytic activity and optimal activity conditions. Furthermore, the hydrolysis characteristics and antioxidant activity of soy and whey protein hydrolyzates obtained through the enzymatic hydrolysis of silkworm thorn fruit were evaluated.

## 2. Results and Discussion 

### 2.1. Proteolytic Characteristics of ESF

#### 2.1.1. Protein Recovery Rate and Proteolytic Activity

**Figure 1:**
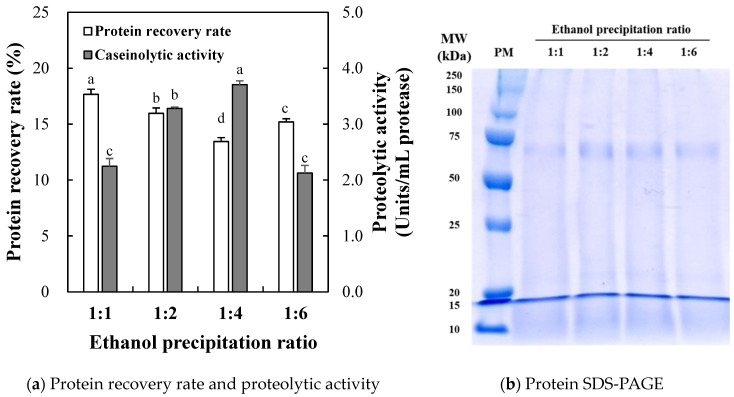
Protein recovery rate and proteolytic activity (**a**) of silkworm thorn (*Cudrania tricuspidata*) fruit fraction precipitated by different ethanol ratios (water-soluble fraction of SF suspension:ethanol) and the protein pattern (**b**) of the ethanol precipitates. SF suspension was prepared by dissolving the freeze-dried SF powder in DDDW at 5% (*w/v*). Each error bar indicates the standard deviation. Bars a–d show means with the same letter within each trait are not significantly at *p* < 0.05 by Tukey’s multiple range test. In the representative SDS-PAGE photo, the protein concentration of 40 μg was loaded through 5% stacking gel and 12% separating gel. PM, standard protein marker.

The protein recovery rate and caseinolytic activity of ESF obtained through different ethanol precipitation ratios of 1:1, 1:2, 1:4, and 1:6 (*v/v*) are shown in Figure 1a. The protein content of ESF ranged from 2.90 to 3.31 mg/mL. The different ethanol precipitation ratios significantly affected the protein recovery rate of ESF. As the ethanol ratio increased from 1:1 to 1:4 ratio, the protein recovery rate of ESF decreased (*p* < 0.05). However, the 1:6 ratio (15.2%) showed a significantly higher protein recovery rate than the 1:4 ratio (13.4%). As a result, the lowest protein recovery rate was found at the 1:4 ratio, indicating that the lowest amount of hydrophilic proteins was collected. 

In general, protein solubility is determined by its interactions with water molecules, and the pH and polarity of solvents play vital roles in these interactions [16]. In this study, no pH differences (pH 7.2–7.6) in ESF solution with different ethanol precipitation ratios were observed (*p* > 0.05). Ethanol precipitation is a practical method used for isolating and purifying proteinous compounds from natural plant sources, in which a decrease in polarity in a protein solution can decrease protein solubility, leading the solubilized hydrophilic proteins to precipitate out of the solution [17]. However, it has been reported that the treatment of polyphenol-rich proteins with non-polar solvents showed inconsistent with the results of general ethanol precipitation [18]. In this regard, a decrease in polarity would release the polyphenols from the non-covalent interactions with proteins, causing an increase in protein solubility [19]. The ripened fruit of silkworm thorns have been well known to have a considerable number of polyphenols (19.7 mg%, dry matter) [20]. Moreover, the higher protein recovery rate at 1:6 compared to 1:4 would be explained by the fact that specific low polarity can interact with the intramolecular hydrophobic sites of protein structure, causing a decrease in protein solubility [19]. Thus, our result shows that the change in protein recovery rate at different ethanol precipitation ratios could be related to the solubility of hydrophilic proteins altered by the protein–polyphenol interaction and the penetration of non-polar solvents into the intramolecular hydrophobic regions.

#### 2.1.2. Proteolytic Activity

The proteolytic activity of ESF, determined by caseinolytic activity, was significantly influenced by the different ratios of ethanol precipitation (Figure 1a). With the different ethanol precipitation ratios, the proteolytic activity of ESF showed opposite trends as compared to the result of the protein recovery rate. This result indicates that ESF at the 1:4 ratio, with the lowest protein recovery rate, presented the highest caseinolytic activity (3.71 Units/mL) (*p* < 0.05). Thus, the major proteolytic fraction may have hydrophilic characteristics, implying that ethanol precipitation could effectively isolate and purify the proteolytic fraction of silkworm thorn fruits. However, the decrease in proteolytic activity at the 1:6 ratio might be related to the dilution effect due to the additional precipitation of other proteins, considering the increased protein content. Previously, Jara et al. [15] noted that the partially purified extract obtained from the latex of *Maclura pomifera* showed 7.2 units/mL of caseinolytic activity and 1.75 mg/mL of protein content. Moreover, the proteolytic activity of plant protease derived from Moraceae fruits might be influenced by the physiological characteristics of plants and the growing environmental conditions since proteolytic enzymes are involved in several protein catabolism [11,15]. Rudenskaya et al. [15] reported that isolated and sorbent-purified macluralisin from *Maclura pomifera* fruits showed an increased purification factor of up to 64-fold. Thus, the impacts of the biological characteristics of the fruit and the isolation and purification process on the proteolytic activity of silkworm thorn fruits would be further needed for its practical use as a commercial plant protease.

#### 2.1.3. SDS-PAGE

The representative photo of the protein SDS-PAGE of the ESF with different ethanol precipitation ratios (1:1, 1:2, 1:4, and 1:6 (*v/v*)) is shown in Figure 1b. As a result, distinct protein bands derived from ESF were observed around 65 kDa, 20 kDa, and 10–15 kDa, regardless of the ethanol precipitation ratios. According to previous research, it has been observed that plant-based proteases can identify polysaccharides existing in fruit peels within the 10–15 kDa range [21]. Since the SF used in our study also includes the peel of a silkworm thorn, the protein band at 10–15 kDa is presumed to be polysaccharides contained in the peel of the silkworm thorn fruit. Serine proteases isolated from plants typically exhibit a molecular weight ranging from 60 to 70 kDa [22]. A previous study has shown that macluralisin, a proteolytic enzyme isolated from *Maclura pomifera* fruit, possesses a protein molecular weight of 65 kDa [23]. In addition, Rudenskaya et al. [11] confirmed that the molecular weight of macluralisin from *Maclura pomifera* fruits corresponded to 67 kDa. To our knowledge, information on the protein profile in silkworm thorn fruit has been limited. Although a detailed comparison of the overall protein pattern is complicated, specific molecular weight distributions can be observed for isolated protein fractions of silkworm thorn fruit under ethanol precipitation conditions. Moreover, this current study suggests that a proteolytic enzyme, presumed to be a macluralisin, may be present in the silkworm thorn fruit, which belongs to the same Moraceae family.

#### 2.1.4. Effect of pH, Temperature, and Inhibitor on Proteolytic Activity 

**Figure 2:**
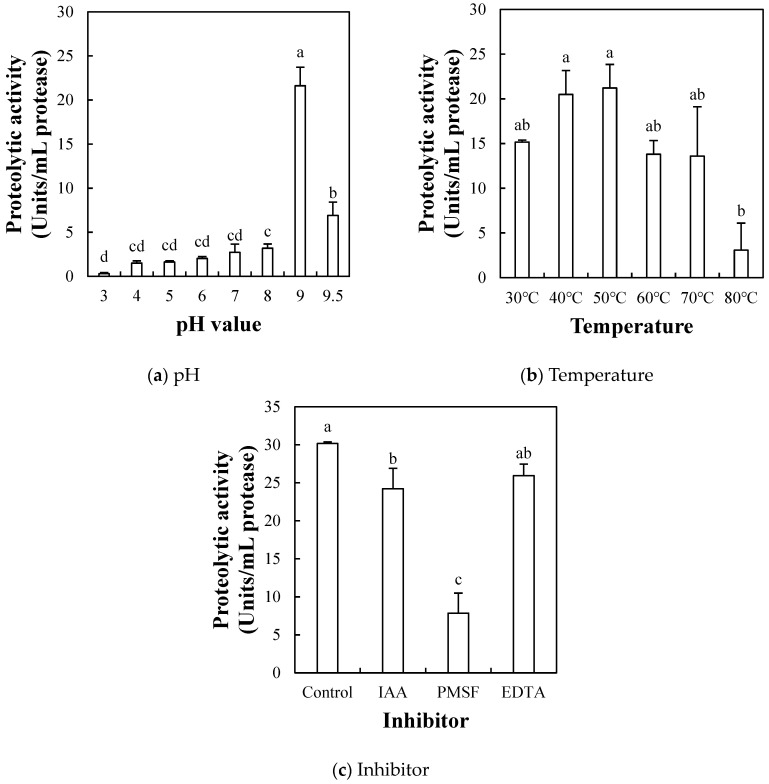
Effects of pH (**a**), temperature (**b**), and inhibitor (**c**) on the proteolytic activity of silkworm thorn (*Cudrania tricuspidata*) fruit fraction precipitated with ethanol at 1:4 ratio (water-soluble fraction of SF suspension:ethanol). SF suspension was prepared by dissolving the freeze-dried SF powder in DDDW at 5% (*w/v*). Each error bar indicates the standard deviation. Bars a–d show means with the same letter within each trait are not significantly at *p* < 0.05 by Tukey’s multiple range test.

To explore the optimal activity conditions and the protease classification, the effects of pH, temperature, and inhibitors on the proteolytic activity of ESF at the 1:4 ratio, showing the highest proteolytic activity, were evaluated (Figure 2a–c). The proteolytic activity based on pH ranged from 0.30 to 16.72 units/mL, with the highest proteolytic activity observed at pH 9.0 (*p* < 0.05). The proteolytic activity of ESF at the 1:4 ratio exhibited consistent activity below 5.0 units/mL within the pH range of 3.0 to 8.0. However, a rapid increase of 16.72 units/mL was observed at pH 9.0. In addition, as the pH increased from 9.0 to 9.5, the proteolytic activity decreased sharply to 6.37 units/mL; thus, the optimum activity of ESF at the 1:4 ratio might be around pH 9.0.

The proteolytic activity based on the temperature change ranged from 3.07 to 21.21 units/mL (Figure 2b), with the highest activity (21.21 Units/mL) observed at 50 °C (*p* < 0.05). The proteolytic activity of ESF at the 1:4 ratio gradually increased up to 50 °C, followed by a sharp decline in activity to 3.07 units/mL at temperatures exceeding 80 °C (*p* > 0.05). According to previous studies, the commercial enzyme Alcalase retains its activity up to 55–60 °C but shows a rapid decrease in activity above 70 °C, whereas papain remains active between 60 and 80 °C [24,25,26]. The findings of this study indicate that the proteolytic activity of ESF at the 1:4 ratio might be stable below 80 °C, suggesting that ESF may have a broad range of effective temperatures for applications in the food industry.

In order to establish the protease classification of ESF at the 1:4 ratio, the typical three inhibitors, IAA (cysteine protease inhibitor), PMSF (serine protease inhibitor), and EDTA (metalloprotease inhibitor), were used (Figure 2c). The proteolytic activity of ESF was almost inhibited by PMSF treatment at the selected concentration of 2 mM. The proteolytic activity slightly inhibited by IAA and EDTA were 24.24 and 25.93 units/mL, respectively (*p* < 0.05). However, the measured proteolytic activity inhibited by PMSF was 7.86 units/mL, indicating that the major proteolytic enzymes in ESF could belong to the serine protease family. Given the incomplete inhibition by PMSF, it could be speculated that the ESF likely contains other proteases beyond serine proteases, in addition to an enzyme presumed to be macluralisin.

Taken together, a fraction exhibiting the most superior proteolytic activity, ESF at the 1:4 ratio, might have proteolytic enzymes with optimal activity at pH 9.0 and 50 °C. In a previous study, the proteolytic enzymes isolated from *Maclura pomifera* were identified as macluralisin and pomiferin. Macluralisin, a serine protease with a molecular weight of 65 kDa, exhibited optimal activity at pH 8.5 and 58 °C. Pomiferin, another serine protease with a molecular weight ranging from 62.7 to 71.2 kDa, exhibited optimal activity at pH 9.2–10.8 and thermal stability at 55 °C [27,28]. Given that the ESF in this study demonstrated optimal activity at pH 8.5 and 85 °C, the proteolytic fraction in ESF may potentially contain proteolytic enzymes such as macluralisin or pomiferin.

### 2.2. Hydrolysis and Antioxidant Characteristics of Protein Hydrolyzate Using SF Powder

Recently, various microbial proteases have been applied increasingly to enhance the production efficiency of human-grade bioactive peptides [29], and the bioactive peptides have been extensively used in the nutraceutical and pharmaceutical industries. In particular, SPI and WPC are considered economical and valid sources for producing bioactive peptides; it has been reported that their enzymatic hydrolyzates can provide several physiological benefits, generally including antioxidant activity [30,31]. Thus, this study determined the hydrolysis and antioxidant characteristics of SPI and WPC hydrolyzates using silkworm thorn fruit powder.

#### 2.2.1. Protein SDS-PAGE

**Figure 3:**
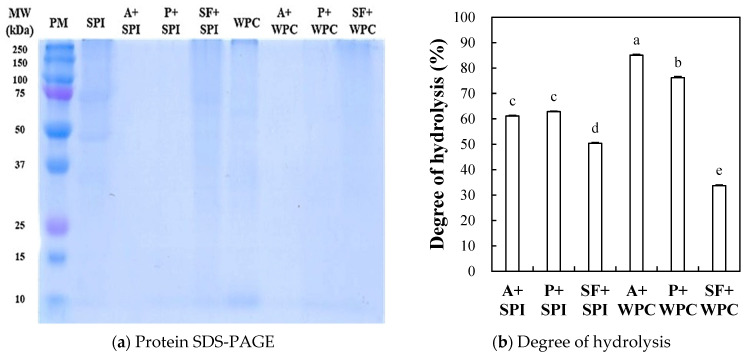
Protein pattern (**a**) and degree of hydrolysis (**b**) of soy protein isolate (SPI) and whey protein concentrate (WPC) hydrolyzates treated by Alcalase (A), papain (P), and silkworm thorn (*Cudrania tricuspidata*) fruit (SF). The enzyme to substrate ratio was fixed as 1% (*w/w*), and the enzymatic hydrolysis was performed at 37 °C for 5 h. Each error bar indicates the standard deviation. Bars a–e showing means with the same letter within each trait are not significantly at *p* < 0.05 by Tukey’s multiple range test.

The representative photo of the protein SDS-PAGE of the SPI and WPC hydrolyzate mediated with Alcalase, papain, and SF is shown in Figure 3a. Major protein bands of soy proteins (50–80 kDa, β-conglycinin; 22–36 kDa, glycinin) [32] and whey proteins (18.3 kDa, β-lactoglobulin; 19.0–25.4 kDa, casein; 80 kDa, lactoperoxidase and lactoferrin) [33] were distinctly visible in SPI and WPC treatments before enzymatic hydrolysis. When Alcalase and papain, commonly used enzymes in the food industry, were employed for the hydrolysis of SPI and WPC, the major protein bands almost disappeared. However, some SPI and WPC protein bands remained in the enzymatic hydrolysis using SF, indicating that SF was less effective in hydrolyzing the proteins contained in SPI and WPC than the two commercial proteases used. In this study, we chose the gel percentage to briefly screen the possibility of hydrolysis of major protein bands contained in SPI and WPC, but further studies would be warranted to identify the small peptides through Tricine-PAGE and MALDI-TOF and to investigate their physiological functionality extensively.

#### 2.2.2. Degree of Hydrolysis (DH)

The degree of hydrolysis (DH) of the SPI and WPC hydrolyzates mediated with Alcalase, papain, and SF powder is shown in Figure 3b. Among the six hydrolyzate treatments, the highest DH (85.07%) was found at A + WPC (WPC hydrolyzed with Alcalase) (*p* < 0.05). The enzymatic hydrolysis using SF resulted in a lower DH in each protein when compared to Alcalase and papain treatments (*p* < 0.05), which was consistent with the result of protein SDS-PAGE (Figure 3a). Improving hydrolysis efficiency is a process aimed at enhancing bioactivity due to the increased generation of peptides and free amino acids. However, an excessive increase in the DH can induce undesirable bitterness in food products [34]. Thus, achieving an appropriate DH is a process to maintain functional properties while not adversely affecting the quality characteristics of the final products. Our results show that the potential for practical utilization of SF as a source for proteolytic enzymes to produce food protein hydrolyzates could be considered.

#### 2.2.3. Antioxidant Activity of SPI and WPC Hydrolyzate

**Figure 4:**
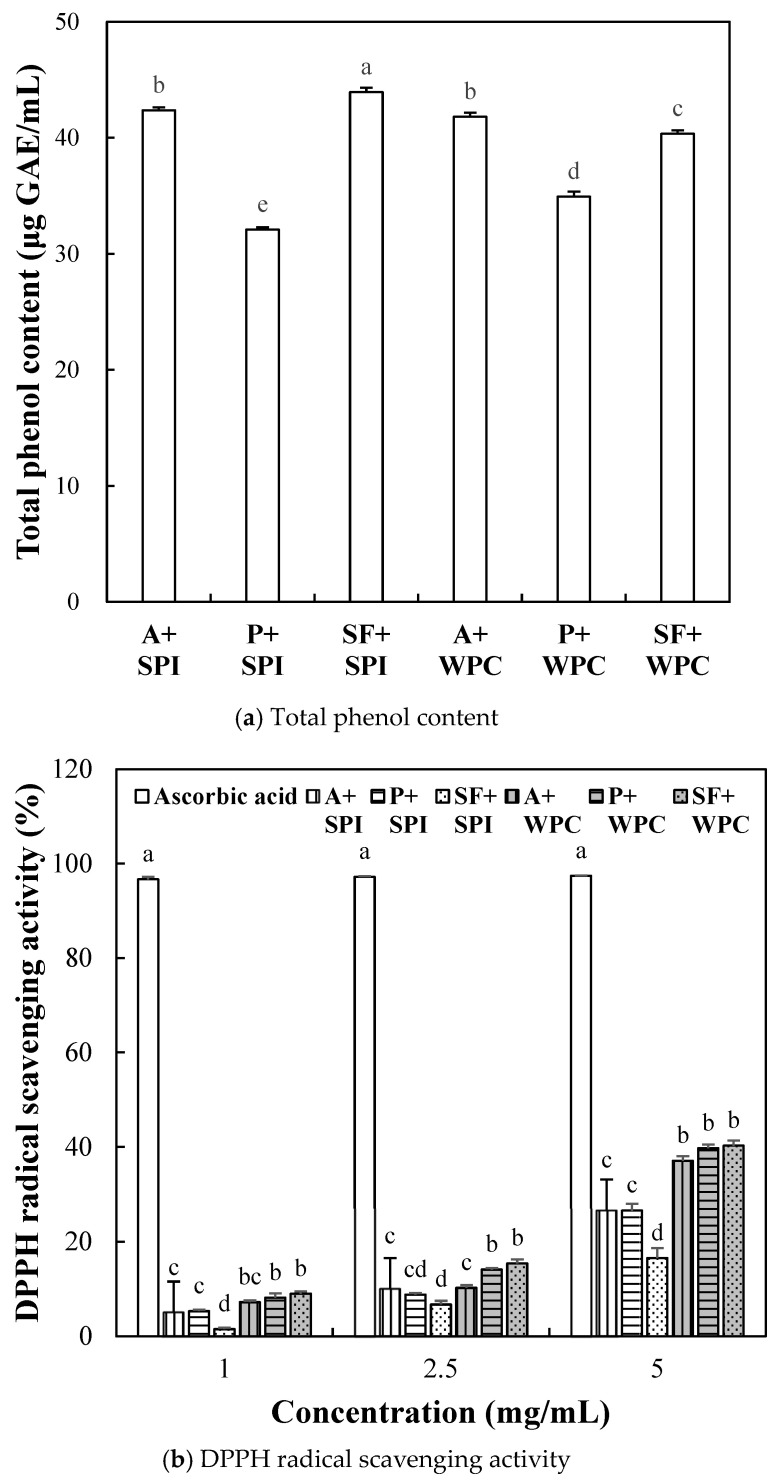

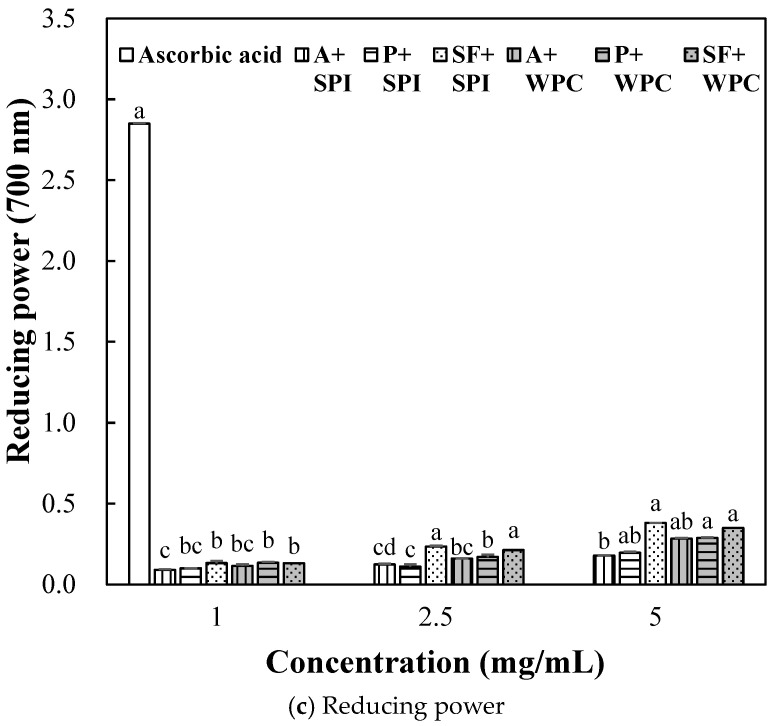
Total phenol content (**a**), 2,2-diphenyl-1-picrylhydrazyl (DPPH) radical scavenging activity (**b**) and reducing powder (**c**) of soy protein isolate (SPI) and whey protein concentrate (WPC) hydrolyzates treated by Alcalase (A), papain (P), and silkworm thorn (*Cudrania tricuspidata*) fruit (SF). The enzyme to substrate ratio was fixed as 1% (*w/w*), and the enzymatic hydrolysis was performed at 37 °C for 5 h. Each error bar indicates the standard deviation. Bars a–e show means with the same letter within each trait are not significantly at *p* < 0.05 by Tukey’s multiple range test.

The total phenol content of the SPI and WPC hydrolyzed with Alcalase, papain, and SF is shown in Figure 4a. WPC possesses antioxidant activity due to the presence of bioactive peptides derived from various enzyme systems, including lactoferrin, ascorbic acid, and β-carotene, whereas SPI exhibits a strong antioxidant activity due to the presence of phenolic components and isoflavones [35]. Phenolic compounds typically form phenol-protein conjugates through non-covalent physical interactions and covalent bonds. According to previous studies, the antioxidant activity increases with the rising concentration of oxidized polyphenols during hydrolysis. This could contribute to increases in antioxidant activities, such as DPPH radical scavenging activity and reducing power [36]. In this study, SF + SPI treatment (SPI hydrolyzed with SF) exhibited the significantly highest total phenolic content (44 μg GAE/mL). A previous study noted that milk contains 3.0 mg GAE/100 g per 100 g, contributing to DPPH radical scavenging activity and reducing power [37], and it has been reported that whey protein concentrate contains well-known antioxidants such as ascorbic acid, α-tocopherol, β-carotene, riboflavin, and thiamin [38]. Additionally, soy protein is a rich source of isoflavones and phenolic compounds, primarily consisting of major phenolic compounds such as gallic acid, ferulic acid, and syringic acid [39]. Jeong et al. [40] show that a silkworm thorn fruit contains 56.42 mg GAE/100 g of phenolic content. In this study, thus, the different total phenol content of SPI and WPC protein hydrolyzates could be associated with the phenolic content of original materials and the different impacts of proteolytic enzymes on protein–polyphenol interaction.

The DPPH radical scavenging activity measured at three different concentrations (1, 2.5, and 5 mg/mL) showed a concentration-dependent increase, regardless of the type of hydrolyzate (Figure 4b). According to a previous study, WPC has shown higher DPPH radical scavenging activity than SPI [41]. Similarly, in our study, the WPC hydrolyzate mediated with SF exhibited the highest DPPH radical scavenging activity at all measured concentrations. Additionally, SF + SPI treatment showed 41.32% DPPH radical scavenging activity compared to the positive control (L-ascorbic acid) at 5 mg/mL concentration. According to Abu-Salem et al. [42], the DPPH radical scavenging activity of SPI hydrolyzates increased up to 70% in some peptide fractions, attributed to the increase in amino acids Leu, His, and the breakdown of β-conglycinin and glycinin in low molecular weight hydrolyzates. In this study, SPI hydrolyzates at a concentration of 5 mg/mL exhibited a maximum DPPH radical scavenging activity of 26.61%, which appears to be relatively lower, probably due to the unseparated and unpurified hydrolyzate used for analysis.

The reducing power measured at three different concentrations showed a concentration-dependent manner in all treatments (Figure 4c). However, the reducing power of six hydrolyzates was considerably lower than L-ascorbic acid (*p* < 0.05). At 2.5 and 5 mg/mL concentrations, SF + SPI and SF + WPC treatments (SPI and WPC hydrolyzed with SF) exhibited significantly higher reducing power than Alcalase and papain treatment. Protein–polyphenol conjugates formed through covalent and non-covalent bonds between proteins and polyphenols exhibit stronger antioxidant activities than the original proteins. Studies have shown that forming these conjugates can enhance the antioxidant activity of protein hydrolyzates [43]. This suggests that the high phenolic content in the SF + SPI treatment (Figure 4a) may have contributed to an increase in protein–polyphenol conjugate formation, thereby enhancing the reducing power of the hydrolyzates.

## 3. Materials and Methods

### 3.1. Raw Material

**Figure 5:**
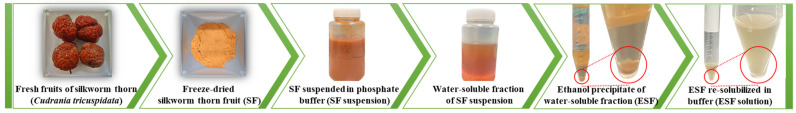
A schematic diagram describing the isolation and partial purification procedure of proteolytic fraction from silkworm thorn (*Cudrania tricuspidata*) fruit.

A schematic diagram describing the isolation and partial purification procedure of the proteolytic fraction from silkworm thorn fruit is shown in Figure 5. The fresh fruits of the silkworm thorn (*Cudrania tricuspidata*) were manually harvested from adult trees grown in a commercial orchard at Sancheong in Korea (latitude: 35°01′ N, longitude: 127°88′ E) in November 2021. The whole fruits (an average weight of 12 g) were quickly washed, frozen in a −80 °C freezer, and freeze-dried using a freeze dryer (MG-VFD100, MG Industrial Ltd., Gunpo, Republic of Korea). The freeze-dried silkworm thorn fruit powder (SF) was pulverized in a commercial food blender, vacuum-packaged in a nylon/polyethylene (NY/PE) bag and stored at −20 °C until further analysis.

### 3.2. Isolation and Partial Purification of Proteolytic Fraction

The proteolytic fraction derived from SF was isolated and partially purified through ethanol precipitation, according to the method of Jara et al. [15]. Ten grams of SF were homogenized with 190 mL of 0.1 M sodium phosphate buffer (pH 6.6) with 5 mM cysteine and 5 mM ethylenediaminetetraacetic acid (EDTA). The SF suspension was placed at 4 °C for 12 h and centrifuged at 3700× *g* for 60 min at 4 °C. The supernatant was collected to isolate water-soluble compounds from the SF suspension, including solubilized hydrophilic proteins. The proteolytic fraction from the supernatant was partially purified through ethanol precipitation (99.9% anhydrous, Samchun Chemical Co., Ltd., Gyeonggi-do, Republic of Korea) at four different ratios (water-soluble fraction of SF suspension:ethanol) of 1:1, 1:2, 1:4, and 1:6 (*v/v*). The supernatant–ethanol mixture was centrifuged at 3700× *g* for 60 min at 4 °C, and the residual ethanol precipitate was collected and re-solubilized in 1 mL of 0.1 M sodium phosphate buffer (pH 6.6) with 5 mM cysteine and 5 mM EDTA. The ethanol fractional precipitate obtained from the water-soluble fraction of SF suspension (ESF) was stored at –20 °C until further analysis.

### 3.3. Proteolytic Characteristics of ESF

#### 3.3.1. Protein Recovery Rate

The protein recovery rate of ESF was calculated based on the percentage difference in protein concentration between the water-soluble fraction of SF suspension before ethanol precipitation (A) and the ESF solution (B). The protein content was measured using the biuret method developed by Feng et al. [44]. The biuret reagent was prepared as follows; 1.5 g of copper sulfate and 6 g of sodium potassium tartrate were solubilized entirely in 500 mL of DDDW, and 300 mL of 10% (*w/v*) sodium hydroxide solution was added. Finally, the volume of the biuret solution was mass up to 1000 mL with additional DDDW. One milliliter of aliquot was reacted with 4 mL of the biuret reagent at room temperature for 20 min, and the absorbance of the sample was read at 540 nm. The protein content was calculated using the standard curve of bovine serum albumin (BSA) and expressed as milligrams of protein per milliliter sample (mg/mL). The protein recovery rate was calculated according to Formula (1).

Protein recovery rate (%) = (protein concentration of B (mg/mL)/protein concentration of A (mg/mL)) × 100.(1)

#### 3.3.2. Proteolytic Activity

The caseinolytic activity of ESF was measured to determine its proteolytic activity according to the standard method outlined by the Sigma Chemical Company [45], as described by Ahmmed et al. [46]. Initially, 0.65% (*w/v*) casein (casein from milk, Samchun Chemical Co., Ltd., Gyeonggi-do, Republic of Korea) was dissolved in 50 mM potassium phosphate buffer (pH 7.5), and the casein solution was incubated at 37 °C for 10 min to achieve equilibrium. Subsequently, 100 μL of ESF solution diluted to the same protein concentration (2 mg/mL) was mixed with 1.7 mL of the casein solution and incubated at 37 °C for 10 min. To stop the reaction, 500 μL of 110 mM trichloroacetic acid (TCA) solution was added and centrifuged at 21,000× *g* for 20 min. A total of two hundred microliters of the supernatant were carefully transferred into a clean plastic tube, and 500 μL of 500 mM sodium carbonate and 100 μL of 0.5 M Folin-Ciocalteu’s phenol reagent (F9252, Sigma-Aldrich, St. Louis, MO, USA) were added to the tube. The sample mixture was incubated at 37 °C for 30 min, and the incubated sample was centrifuged at 21,000× *g* for 20 min. Lastly, 200 μL of the supernatant was transferred to a 96-well plate, and the sample absorbance was read at 660 nm using a microplate reader (Synergy H1, BioTek, Winooski, VT, USA). The proteolytic activity of the sample was calculated using a regression equation of y (μM tyrosine equivalents) = 2.5214 (Absorbance) x + 0.0127 (*R*^2^ = 0.999), which was provided by plotting the absorbance against the serial concentrations of tyrosine standard solutions (0, 0.05, 0.1, 0.2, 0.4, and 0.6 μM). The caseinolytic activity, quantified regarding tyrosine equivalent units, was calculated according to Formula (2).

(2) $$\mathrm{Units}/\mathrm{mL}\;\mathrm{protease}=\frac{\mu mol\;tyrosine\;equivalents\times VT}{VE\times T\times VA}$$

where,

VT = total volume (mL) of assay.
VE = volume of protease (mL).
T = time of assay (min) as per the unit definition.
VA = volume (mL) used in colorimetric determination.

#### 3.3.3. Sodium Dodecyl Sulphate Polyacrylamide Gel Electrophoresis (SDS-PAGE)

Protein SDS-PAGE was performed using the Laemmli (1970) [47] method to investigate the protein molecular weight of ESF. The ESF solutions diluted to the same protein concentration (2.5 mg/mL) were mixed with a sample buffer (60 mM Tris-HCl (pH 6.8), 25% glycerol, 2% SDS, 14.1 mM β-mercaptoethanol, 0.1% bromophenol blue) at a 4:1 ratio. The sample mixture was denatured at 100 °C for 5 min in a constant-temperature water bath (thermo beth ALB128, FINEPCR, Gunpo-si, Republic of Korea). A total of twenty microliters (40 μg protein loaded) of the heat-denatured sample mixture were loaded into the electrophoresis system (mini protein tetracell, Bio-Rad, Hercules, CA, USA) into 12% polyacrylamide gel (5% stacking gel and 12% separating gel). The loaded sample passed through the stacking gel at 80 V for approximately 10 min and then passed through the separating gel at 120 V. The gel was stained with staining solution (0.25% (*w/v*) Coomassie blue R-250, 50% (*v/v*) methanol, 40% (*v/v*) distilled water, and 10% (*v/v*) acetic acid) and decolorized with a de-staining solution (50% (*v/v*) methanol, 40% (*v/v*) distilled water, and 10% (*v/v*) acetic acid). A protein standard marker (Precision plus protein standards, 1610373, BIO-Rad, Hercules, CA, USA) was used to determine the molecular weight.

#### 3.3.4. Effects of pH, Temperature, and Inhibitor on Proteolytic Activity

The effects of pH, temperature, and inhibitor on the proteolytic activity of the ESF (1:4 ratio), which showed the highest proteolytic activity among different ethanol precipitation ratios (Figure 1), were determined [48]. To determine the optimal pH and temperature, caseinolytic activity was determined at different pH values (3.0, 4.0, 5.0, 6.0, 7.0, 8.0, 9.0, and 9.5) and temperatures (30 °C, 40 °C, 50 °C, 60 °C, 70 °C, and 80 °C). The following buffers were used to form each targeted pH: 50 mM sodium citrate buffer (pH 3.0–6.0), 50 mM sodium phosphate buffer (pH 7.0), and 50 mM Tris-HCl buffer (pH 8.0–9.5). The optimum temperature for proteolytic activity, the reaction mixture was incubated at each selected temperature, and the caseinolytic activity was determined at 37 °C. To determine the effect of protease inhibitors iodoacetamide IAA (IAA, cysteine protease inhibitor, Sigma Aldrich-St. Louis, MO, USA), phenyl methyl sulphonyl fluoride (PMSF, serine protease inhibitor, Sigma Aldrich-St. Louis, MO, USA) and EDTA (metalloprotease inhibitor, Samchun Chemical Co., Ltd., Gyeonggi-do, Republic of Korea) were used at the same concentration of 2 mM. The supernatant of sample solutions diluted with tris-buffered saline (TBS, ELPIS Biotech, Daejeon, Republic of Korea) with inhibitors were pre-incubated for 30 min at room temperature. Subsequently, the pre-incubated sample was measured following the procedure outlined in Section 3.3.2 on proteolytic activity.

### 3.4. Enzymatic Hydrolysis of Soy and Whey Proteins

To explore further the commercial applications on food protein hydrolysis, extensively used food proteins for generating protein hydrolyzates, soy protein isolate (SPI, 82–83 g/100 g of protein content, VB FOOD Co., Ltd., Seoul, Republic of Korea) and whey protein concentrate (WPC, 82.6 g/100 g of protein content, Sewoo Co., Ltd., Gwng-ju, Republic of Korea), were enzymatically hydrolyzed by commercially available enzymes, Alcalase 2.4 L (a declared activity of 2.4 AU/kg, the density of 1.18 g/mL, Novozymes, Bagsværd, Denmark), papain (from papaya latex, 1.5–10 Units/mg solid, Sigma Aldrich-St. Louis, MO, USA), and SF powder (mentioned in 2.1. raw material). Initially, 10% (*w/v*) SPI and WPC solutions were individually prepared in DDDW, and three aliquots were separated from each protein solution. The enzyme-to-substrate-ratio (E:S) was fixed at 1%, and each proteolytic enzyme was added to achieve the targeted E:S. The mixture was homogenized at 1000 rpm for 60 s using a homogenizer (HG-15A, Daihan Sci., Seoul, Republic of Korea). The pH of the substrate/enzyme mixture was as follows: Alcalase + SPI, 6.7; papain + SPI, 6.6; SF + SPI, 6.6; Alcalase + WPC, 6.5; papain + WPC, 6.4; SF + WPC, 6.5. In this study, pH adjustment for allowing optimal pH of each proteolytic enzyme was not considered to minimize the use of chemicals. Nevertheless, the pH range of the substrate/enzyme mixture was close to or belonged to the optimal pH range of the three proteolytic enzymes (pH 7–9 of Alcalase, pH 5.8–7.0 of papain, and pH 7.5–9.0 of macluralisin). The enzymatic hydrolysis process was conducted by continuously stirring in a constant-temperature water bath at 37 °C for 5 h. After hydrolysis, the mixture was heated to 90 °C for 20 min to inactivate the proteolytic activity. The protein hydrolyzates of SPI and WPC were then cooled at room temperature for 30 min and subsequently filtered through a test sieve (885705, CHUNG GYE GONG SA, Seoul, Republic of Korea) with a mesh size of 100 μm and wire diameter of 71 μm. The filtrated SPI and WPC hydrolyzates were stored at 4 °C for further analysis.

### 3.5. Hydrolysis and Antioxidant Characteristics of SPI and WPC Hydrolyzates

#### 3.5.1. SDS-PAGE

A protein SDS-PAGE of the SPI and WPC hydrolyzates was performed using the method of Laemmli (1970) [47]. The procedure was carried out in the same manner as described in Section 3.3.3. The protein concentration of the loaded sample was equally fixed at 5 mg/mL. A total of twenty microliters (80 μg protein) of the heat-denatured sample mixture were loaded into the electrophoresis system. The standard marker was used to determine the molecular weight.

#### 3.5.2. Degree of Hydrolysis (DH)

The degree of hydrolysis (DH) of the SPI and WPC hydrolyzates generated through the enzymatic hydrolysis of Alcalase, papain, and SF was determined by the Pierce™ BCA Protein Assay Kits (23225#, thermoscientific, Rockford, IL, USA) standard analysis method [49]. A total of two milliliters of each hydrolyzate sample and 2 mL of 20% (*w/v*) TCA were mixed and centrifuged at 1600× *g* for 30 min. According to the bicinchoninic acid (BCA) method, 30 μL of the supernatant was then dispensed into a 96-well plate, and 200 μL of a solution (BCA solution A, and B at 49:1 (*v*/*v*) ratio) was dispensed and reacted at 37 °C for 30 min. The absorbance of the mixture was measured at 560 nm. The protein content was calculated using a standard curve of bovine serum albumin (BSA), and the DH was calculated according to Formula (3) as follows:

DH (%) = 10% TCA soluble protein (mg)/Total protein (mg) × 100.(3)

where total protein indicates the protein concentration of the sample before enzymatic hydrolysis, TCA soluble protein indicates the protein concentration of the sample after enzymatic hydrolysis and TCA precipitation.

#### 3.5.3. Total Phenol Content

The total polyphenol content of the SPI and WPC hydrolyzates was evaluated using the Folin method [50]. Overall, one milliliter of the diluted sample (1 mg/mL), 1 mL of Folin-Ciocalteu’s phenol reagent, and 1 mL of 10% (*w/v*) sodium carbonate solution was mixed. The mixture was allowed to react at room temperature for 1 h before the absorbance was measured at 700 nm. The total polyphenol content of the samples was calculated using a standard calibration curve of gallic acid (GAE) and expressed as gallic acid equivalents per mL of sample (μg GAE/mL).

#### 3.5.4. 2,2-Diphenyl-1-picrylhydrazyl (DPPH) Radical Scavenging Activity

The 2,2-diphenyl-1-picrylhydrazyl (DPPH) radical scavenging activity of the SPI and WPC hydrolyzates was evaluated by the method of Sun et al. [51]. A total of two milliliters of diluted hydrolyzate samples (1, 2.5, and 5 mg/mL) and 2 mL of 0.1 mM DPPH solution dissolved in 95% ethanol were mixed and kept in a dark room for 30 min. The absorbance of the sample was measured at 517 nm. A_blank_ was an absorbance measured by mixing 2 mL of the sample with 95% ethanol, and A_control_ was an absorbance measured by mixing 2 mL of 95% ethanol and DPPH solution. Serial diluted L-ascorbic acid (Daejung Co., Ltd., Gyeonggi-do, Republic of Korea) (1, 2.5, and 5 mg/mL) was used as a positive control. The DPPH radical scavenging activity was calculated according to Formula (4) as follows:

DPPH radical scavenging activity (%) = [1 − (A_sample_ − A_blank_)/A_control_] × 100. (4)

#### 3.5.5. Reducing Power

The reducing power of the SPI and WPC hydrolyzates was carried out by the method of Athukorala et al. [52]. A total of two hundred and fifty microliters of diluted sample (1, 2.5, and 5 mg/mL) were mixed with 250 μL of 0.2 M phosphate buffer (pH 6.6) and 250 μL of 1% (*w/v*) potassium ferricyanide, followed by 20 min incubation at 50 °C. After the reaction, 0.25 mL of 10% (*w/v*) TCA was added, and after taking a 0.25 mL aliquot, it was mixed with 0.25 mL of distilled water and 2 mL of 0.1% ferric chloride. The mixture was then reacted for 10 min at 37 °C, and the absorbance was measured at 700 nm. The reducing power was determined by the difference in absorbance values between samples, with the positive control represented by the reducing power of 1, 2.5, and 5 mg/mL concentrations of L-ascorbic acid measured using the same method.

### 3.6. Statistical Analysis

All data were expressed as mean ± standard error (S.E) (*n* = 3). Data from all measured variables were analyzed using the general linear model (GLM) procedure in the SPSS 18.0 program (SPSS Inc., Chicago, IL, USA). In the variables with statistically significant effects at 5% critical value, Tukey’s multiple range test was used to determine the significance of the differences between treatments (*p* < 0.05).

## 4. Conclusions

In conclusion, the ethanol precipitation ratios significantly influenced the protein recovery rate and proteolytic activity of fractional ethanol precipitate obtained from the water-soluble fraction of silkworm thorn fruits. The proteolytic activity, determined by caseinolytic activity, showed an inverse trend with the protein recovery rate, and the optimal proteolytic activity around pH 9.0 and 50 °C, possibly containing proteolytic enzymes, such as macluralisin or pomiferin, that are commonly found in Moraceae fruits. In the application phase, when compared to Alcalase and papain extensively used as commercial enzymes, silkworm thorn fruit powder was less effective in hydrolyzing SPI and WPC. Nevertheless, SPI and WPC hydrolyzates mediated with silkworm thorn fruit powder showed even better antioxidant activities compared to those mediated with Alcalase and papain. Considering the high-thermal stability up to 80 °C, moreover, our results show that the potential application of the silkworm thorn fruit as a novel source of a plant protease for producing human-grade protein hydrolyzates. For industrial application, further studies determining (1) the identification of proteolytic enzymes contained in silkworm thorn fruit, (2) optimization of the isolation and purification process of the proteolytic enzymes, and (3) the physiological functionality evaluation of specific peptides produced through the proteolytic enzyme of silkworm thorn fruits would be warranted.

## Data Availability

The data presented in this study are available on request from the corresponding author.
